# Evaluating the impact of 3-nitrooxypropanol supplementation on enteric methane emissions in pregnant nonlactating dairy cows offered grass silage

**DOI:** 10.3168/jdsc.2024-0591

**Published:** 2024-08-16

**Authors:** B. Lahart, L. Shalloo, C. Dwan, N. Walker, H. Costigan

**Affiliations:** 1 Teagasc, Animal and Grassland Research and Innovation Centre, Moorepark, Fermoy, Co. Cork, Ireland, P61 P302; 2 School of Biological, Earth and Environmental Sciences, University College Cork, Cork, Ireland T12 K8AF; 3 DSM Nutritional Products, Animal Nutrition & Health, 4002 Basel, Switzerland

## Abstract

Although 3-nitrooxypropanol (3-NOP; Bovaer10) has been proven to reduce enteric methane (CH_4_) by ∼30% in indoor systems of dairying when the additive is mixed throughout TMR and partial mixed ration (PMR) diets, there has been limited research to date on the CH_4_ abatement potential of 3-NOP when mixed within a diet based on perennial ryegrass silage only and fed to pregnant nonlactating dairy cows. To investigate the effect of 3-NOP supplementation on enteric CH_4_ emissions of pregnant nonlactating dairy cows, a 6-wk study was undertaken in which treatment cows were supplemented with 3-NOP mixed within grass silage, whereas control cows were offered grass silage without additive supplementation. Enteric CH_4_, hydrogen (H_2_), and carbon dioxide (CO_2_) were measured using a GreenFeed machine. Body weight, BCS, DMI, and calf birth weight were monitored to determine the effect of 3-NOP supplementation on productivity. The measured dietary concentration of 3-NOP was 63 (range 53.2–77.1) mg/kg DM. Cows supplemented with 3-NOP produced 22% less enteric CH_4_ per day and CH_4_ per kilogram of DMI, respectively, than cows offered the control diet. Cows supplemented with 3-NOP also produced 4.4-fold more H_2_ and H_2_ per kilogram of DMI, respectively, relative to the control. The 3-NOP supplementation had no impact on DMI, BW, BCS, or calf birth weight. Supplementing pregnant nonlactating dairy cows with 3-NOP during the winter housing period effectively reduced enteric CH_4_ emissions without detriment to productivity.

## Highlights

• Supplementation with 3-NOP reduced the enteric methane output of nonlactating dairy cows by 22%.
• 3-NOP increased enteric hydrogen emissions by 4.4-fold.
• 3-NOP supplementation had no impact on dry matter intake, body weight, body condition score, or calf birth weight.

## Graphical Abstract

**Figure:**
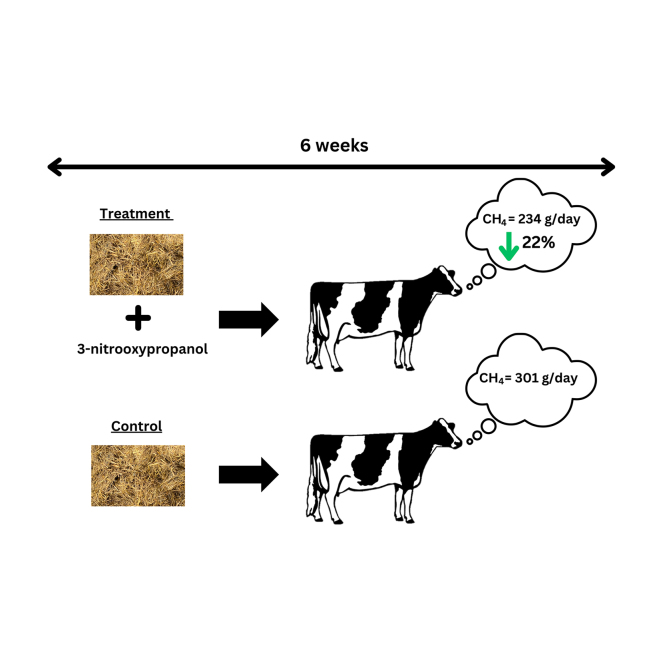
**Summary:** This study evaluated the potential of the feed additive 3-nitrooxypropanol (3-NOP) to reduce enteric methane emissions of nonlactating dairy cows when mixed throughout a perennial ryegrass silage-based diet.

Methane emissions from enteric fermentation account for the majority of greenhouse gas emissions from dairy production systems in Ireland (EPA, 2023). Feed additives provide a solution to reduce enteric CH_4_ output (Beauchemin et al., 2022). At present, 3-nitrooxypropanol (**3-NOP**; Bovaer10; 10% 3-NOP on carrier) is one of the most promising antimethanogenic feed additives internationally (Beauchemin et al., 2022), with reductions in CH_4_ of 33% reported when offered within TMR and partial mixed ration (**PMR**) diets (Kebreab et al., 2023). Hristov et al. (2015) postulated that 3-NOP is more effective in such diets, because the additive is mixed throughout the feed and thereby ingested at regular intervals during the day. In seasonal calving pasture-based dairy systems located in temperate regions such as Ireland, climatic conditions allow for the majority of a dairy cow's diet to come from grazed grass (O'Brien et al., 2018). Supplementing dairy cows with feed additives, such as 3-NOP, at pasture is considerably more difficult and less effective compared with indoors as the only practical opportunities for supplementation are twice daily at morning and evening milking (Costigan et al., 2024). However, during their nonlactating period, seasonal calving pasture-based dairy cows in temperate regions are generally housed indoors over the winter months due to inclement weather conditions and offered grass silage (Dillon et al., 1995). This housing period may present an opportunity to substantially reduce CH_4_ output if 3-NOP is mixed throughout the grass silage offered. Research has demonstrated 3-NOP supplementation to be effective when mixed within a grass silage–based diet and offered to lactating dairy cows (van Gastelen et al., 2022). However, the efficacy of 3-NOP is influenced by various dietary and animal characteristics (Dijkstra et al., 2018), meaning results should not be extrapolated across differing systems. At present, there is limited information on the impact of 3-NOP supplementation when offered in grass silage to nonlactating dairy cows. Therefore, the objective of the present study was to evaluate the impact of 3-NOP (Bovaer10; 10% of 3-NOP; DSM Nutritional Products Ltd., Kaiseraugst, Switzerland) on the CH_4_ output of pregnant nonlactating dairy cows offered grass silage. The hypotheses of the experiment was that 3-NOP supplementation would reduce CH_4_ output in pregnant nonlactating dairy cows offered grass silage.

The current study was conducted in Teagasc Moorepark over a 6-wk period between mid January and late February 2023. The experiment was under the approval of the Teagasc Animal Ethics Committee (TAEC2352–2022) and was conducted in accordance with the Cruelty to Animals Act (Ireland 1876, as amended by European Communities regulations 2002 and 2005) and the European Community Directive 86/609/EC. Thirty-two multiparous, pregnant nonlactating dairy cows with a mean dry-off date of December 11, 2022 (SD = 12 d), were assigned to the study. The cows were divided into 2 homogeneous treatment groups (n = 16 per group) using a balanced randomization procedure based on parity (1, 2, and 3+), breed (Holstein-Friesian and Holstein-Friesian × Jersey crossbred), expected calving date (March 21, 2023 ± 11.8 d), BW (602 ± 46.9 kg), and CH_4_ output (258 ± 36.3 g/d). After this step, the groups were randomly assigned to 1 of 2 treatments: control and treatment. Cows were fed once daily using a Keenan mixing wagon (Keenan, Richard Kenan & Co. Ltd., Borris, Co. Carlow, Republic of Ireland). The control cows solely received perennial ryegrass (**PRG**) silage, whereas treatment cows received PRG silage with 3-NOP. The 3-NOP was mixed with the silage using a “farm pack.” The farm pack included wheat flour (210,000 mg/kg), calcium carbonate (110,000 mg/kg), sepiolite (600,000 mg/kg), and 3-NOP (8,000 mg/kg). Supplementing 3-NOP through a farm pack is reflective of the way in which 3-NOP would be fed at the commercial farm level. The target inclusion rate of the farm pack was 10.5 g of material per kg of silage DM to ensure the target inclusion of 80 mg of 3-NOP per kg of total dietary DM. The farm pack was mixed with the silage for a 5-min period before feed out, with treatment cows always fed directly after the control cows. After treatment cows had been fed, all other cows on the farm (i.e., those not assigned to the experiment) were fed a rinsing diet so that the mixing wagon would be cleaned out before feeding the following day.

Body weight was measured weekly (using electronic weighing scales (Tru-Test Ltd., Auckland, New Zealand) and scales were calibrated before use using known weights. Body condition score was also assessed on a weekly basis on a scale of 1 to 5 (1 = emaciation and 5 = obesity; Edmonson et al., 1989). Body weight and BCS data were not available in wk 5 of the study due to technical difficulties. Daily individual feed intake was recorded using electronic controlled roughage intake control system feed bins (Hokofarm Group B.V., Marknesse, the Netherlands). The experimental diets were distributed to different feed bins, and treatment and control cows had access to the feed bins containing their respective diets. The cows were fed ad libitum to achieve approximately 10% refusal levels. Enteric CH_4_, CO_2_, and H_2_ were measured using a GreenFeed emissions monitoring system (C-Lock Inc., Rapid City, SD), as described in detail by Hammond et al. (2015). In brief, cows were offered a small quantity of bait concentrate as an incentive to visit the GreenFeed machine. The GreenFeed machine was programmed to dispense 42 g of concentrate, every 30 s, to a maximum of 6 concentrate drops per visit for each animal. Once a cow reached the maximum number of concentrate drops, they were blocked from the machine for a minimum of 6 h. An extractor fan was used to take a sample of the cow's breath while she was eating, which measured the concentration of CH_4_, CO_2_, and H_2_. The mean visits to the GreenFeed during the experimental period were 3.2 and 2.9 visits per cow per day for the control and treatment, respectively. The average CO_2_ recovery across the experiment was 97.7%. Calf birth weight was recorded immediately after calving using an electronic weighing scales (TruTest Ltd., Auckland, New Zealand).

Composite samples of silage offered to treatment and control cows were taken 5 times per week throughout the experiment. From each sample, 100 g was weighed and oven-dried over a 48-h period at 40°C for DM determination. A subsample was milled, stored, and bulked per week of experiment before chemical analysis. The ash content of the silage was determined by combusting the sample using a muffle furnace at 550°C for 16 h. Crude protein content of the silage was determined using a Leco FP-428 nitrogen analyzer (Leco Australia Pty Ltd., Castle Hill, New South Wales, Australia) as described by Sweeney (1989). Neutral detergent fiber and ADF content of the silage were determined using an Ankom 2000 Fiber Analyzer as outlined by Ankom Technology Corporation (NY) as described by Van Soest et al. (1991). Concentrate samples were taken weekly from the GreenFeed machine and dried at 60°C for 48 h for DM determination. Concentrate samples were subsequently analyzed for ash, CP, NDF, and ADF as described above. Weekly silage and concentrate samples, were also analyzed for gross energy (**GE**) content using a Parr 6050 compensated jacket calorimeter (Parr Instrument Company, Moline, IL). The GE content of the silage and concentrate consumed by the cows was used to calculate GE intake, which was in turn used to determine the proportion of GE intake that was converted to CH_4_ (i.e., the Y_m_ factor). A subsample of the treatment silage was also taken twice weekly throughout the experiment, stored at −20°C, and sent to DSM for 3-NOP concentration analysis as described by Schilde et al. (2021).

Three experimental days of data were not used (1 d in wk 1, 1 d in wk 2, and 1 d in wk 5, respectively), due to technical difficulties with the GreenFeed and electronic feed bins. Spot measurements of CH_4_, CO_2_, and H_2_ for each animal were averaged within each week of the study. Data were not used if animals had less than 10 visits to the GreenFeed in a given week. For inclusion in the analysis, animals had to have a minimum of 4 wk of data across the experimental period. Data from 3 cows (1 treatment and 2 control) were not used in the analysis because they calved 2 wk into the experiment; an additional cow from the control group was not used because of poor head positioning with the GreenFeed throughout the study period, resulting in a low measurement frequency. Data were available on 28 cows (15 treatment and 13 control). Twenty-three of these cows (12 treatment and 11 control) had 6 wk of data, whereas 3 cows (2 treatment and 1 control) had 5 wk of data and 2 (1 treatment and 1 control) had 4 wk of data as a result of these cows calving earlier than anticipated or having poor visits during specific weeks.

Statistical analyses was conducted using SAS (version 9.4; SAS Institute Inc., Cary, NC). Data were centered within breed and parity for pre-experimental CH_4_, CO_2_, H_2_, BW, and BCS. The effects of additive supplementation on gaseous emissions, BW, BCS, and feed intake parameters were analyzed using linear mixed models (PROC MIXED). In all models, cow nested within treatment was included as a random effect, and week was included as a repeated effect. Fixed effects included in the models were treatment, breed, parity, and week. Centered pre-experimental variables were used in models as linear covariates where appropriate. An autoregressive covariance structure was used in the models analyzing gaseous emissions and feed intake data, whereas a compound symmetry covariance structure was used in the models analyzing BW and BCS data. The interaction between treatment and week was tested in all models. Only interaction terms that improved (*P* < 0.05) the fit of the data were retained. The effect of 3-NOP supplementation on calf birth weight was evaluated using a model containing calf sex, dam parity, and treatment as fixed effects. Significant associations in all models were confirmed when *P* < 0.05.

The mean (SD) DM content of the grass silage offered to the treatment and control groups were 29.8% (4.07) and 28.8% (3.67), respectively. The mean (SD) ash, CP, NDF, and ADF of the control silage over the study period was 61 (5.0), 132 (12.4), 469 (41.9), and 296 (24.8) g/kg DM, respectively, whereas the mean (SD) ash, CP, NDF, and ADF of the treatment silage over the study period was 61 (5.0), 129 (19.5), 470 (31.8), and 283 (18.5) g/kg DM, respectively. The mean (SD) GE content of the silage offered to the treatment and control cows was 19.24 (0.307) and 18.98 (0.179) MJ/kg DM, respectively. The mean (SD) ash, CP, NDF, and ADF of the concentrate over the study period was 108 (1.6), 162 (2.0), 253 (5.6), and 163 (9.2) g/kg DM, respectively. The mean (SD) of the GE of the concentrate from the GreenFeed was 16.49 (0.027) MJ/kg DM. The measured concentration of 3-NOP from the grass silage offered to the treatment group averaged 68 (range 56.9–83.1) mg/kg DM and was 63 (range 53.2–77.1) mg/kg DM when adjusted for intake of concentrate from the GreenFeed.

There was no impact (*P* = 0.14) of 3-NOP supplementation on the bait feed concentrate DMI from the GreenFeed (0.76 kg; SE 0.021) compared with the control group (0.81 kg; SE 0.023). Similarly, there was no impact (*P* = 0.70) of 3-NOP supplementation on silage DMI (11.98 kg; SE 0.376) compared with the control group (12.18 kg; SE 0.412). The influence of 3-NOP supplementation on total DMI and enteric gas emissions is presented in Table 1. There was no effect of 3-NOP supplementation on total DMI (inclusive of silage and concentrate DMI from the GreenFeed). Supplementation with 3-NOP led to a 67 g reduction in CH_4_ per cow (*P* < 0.001), a 5.36 g reduction in CH_4_ expressed per unit of DMI (*P* < 0.001), and a 1.5% reduction in the proportion of GE intake lost as CH_4_ (*P* < 0.001). A treatment by week interaction was noted for CH_4_ output and CH_4_ per unit of DMI (*P* < 0.001; Figure 1), whereby the magnitude of the difference between the treatment and control groups changed over the study. Similar observations were noted when CH_4_ was expressed as a proportion of GE intake (results not shown). Hydrogen emissions were 4.4-fold greater in the 3-NOP-supplemented cows (*P* < 0.001). A treatment by week interaction was also observed for H_2_ and H_2_ per unit of DMI (*P* < 0.001; Figure 1), whereby the magnitude of the difference between treatment and control cows changed across the study weeks. There was no impact of 3-NOP supplementation on CO_2_ emissions and CO_2_ per unit of DMI, although a treatment by week interaction was observed for CO_2_ emissions (*P* < 0.010) whereby there was no difference between groups with the exception of wk 5 where 3-NOP was lower in treatment cows compared with the control group (results not shown). Although numerically lower, there was no significant impact (*P* = 0.104) of 3-NOP supplementation on BW (638 kg; SE 5.5) compared with the control group (651 kg; SE 6.1). Similarly, there was no impact (*P* = 0.410) of 3-NOP supplementation on BCS (3.44 units; SE 0.043) compared with the control group (3.39 units; SE 0.049). There was no impact (*P* = 0.46) of 3-NOP supplementation on calf birth weight (38 kg; SE 2.8) compared with the control group (40 kg; SE 2.3).

**Table 1:** Least squares means for DMI, enteric emissions, and enteric emissions per kilogram of DMI, of pregnant nonlactating dairy cows offered a perennial ryegrass silage–based diet supplemented with 3-nitrooxypropanol (3-NOP; Treatment) and cows receiving no supplementation (Control)

Item	Control	Treatment	SEM	*P*-value	
				Treatment	Treatment × week
Total DMI^1^ (kg)	13.0	12.7	0.40	0.66	0.28
Methane (g)	301	234	3.9	<0.001	<0.001
Carbon dioxide (kg)	11.7	11.6	1.57	0.76	0.013
Hydrogen (g)	0.61	3.29	0.198	<0.001	<0.001
Methane/total DMI (g/kg)	23.7	18.4	0.71	<0.001	<0.001
Methane lost as a proportion of GEI^2^ (%)	6.9	5.4	0.20	<0.001	<0.001
Carbon dioxide/total DMI (g/kg)	912	934	28.7	0.58	0.19
Hydrogen/total DMI (g/kg)	0.05	0.27	0.019	<0.001	<0.001

1 Combined DMI of grass silage and concentrate.

2 Gross energy intake.

**Figure 1:**
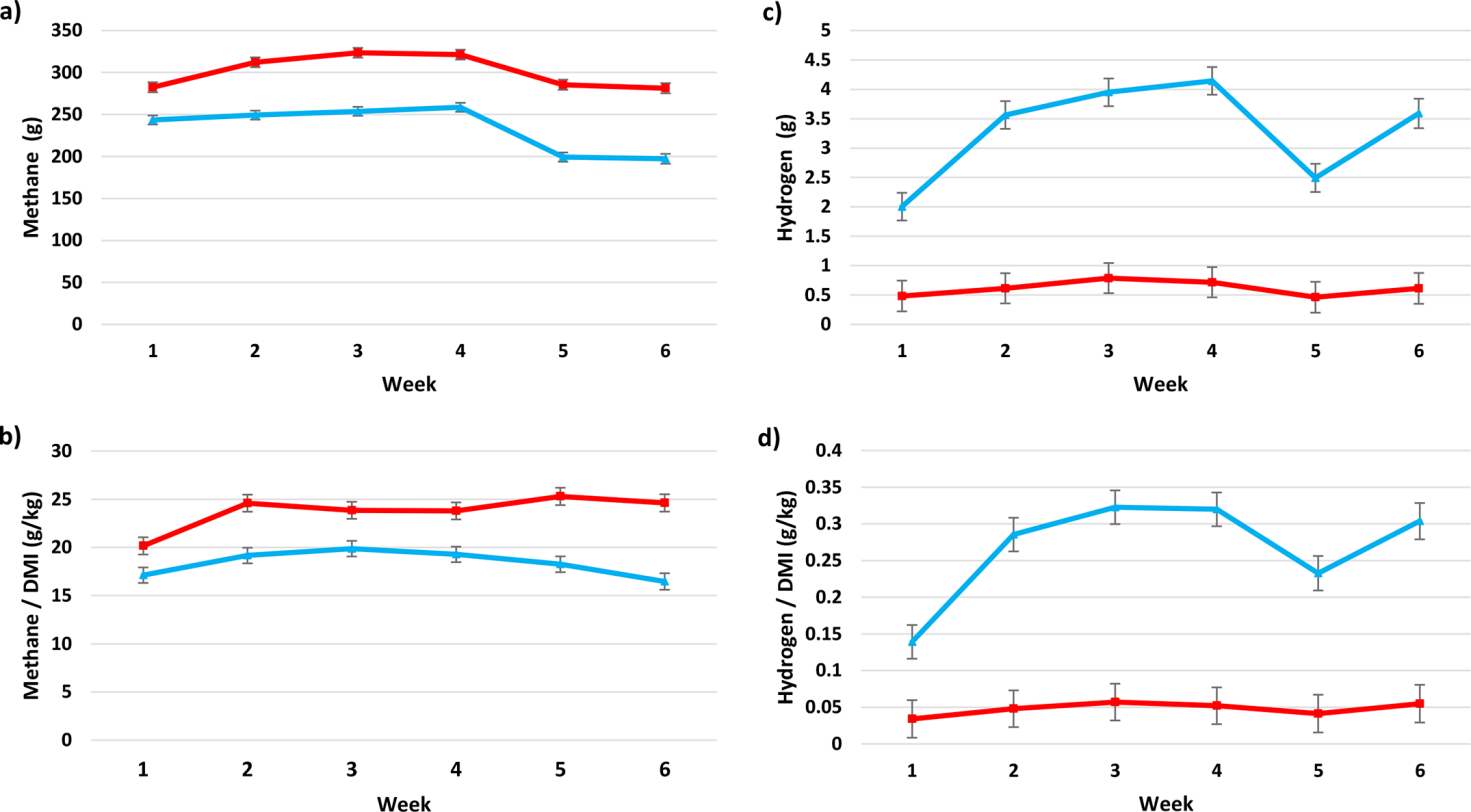
Graphs showing the effect of supplementation with 3-nitrooxypropanol (3-NOP; TRT) on (a) methane emissions, (b) methane per unit of DMI, (c) hydrogen emissions, and (d) hydrogen per unit of DMI of TRT (blue lines) or control cows (red lines). Error bars represent SE.

The antimethanogenic feed additive 3-NOP provides dairy producers with an effective solution to reduce CH_4_ emissions of animals housed indoors. To date, 3-NOP has been extensively studied with lactating dairy cows when offered within high-quality TMR and PMR diets (Kebreab et al., 2023). However, there is limited information on the efficacy of 3-NOP offered to nonlactating dairy cows within grass silage–based diets as practiced in the current study.

Once ingested, 3-NOP binds to the enzyme methyl-coenzyme M reductase within the rumen, which is responsible for catalyzing the last step of methanogenesis (Duin et al., 2016), thus preventing methanogenic archaea from combining CO_2_ with H_2_, and forming CH_4_. Findings in the present study indicate that feeding 3-NOP within a PRG silage diet can achieve substantial reductions in CH_4_ per unit of DMI relative to the control (−22%; Table 1), which is slightly greater than reported by van Gastelen et al. (2024; −16%) with nonlactating dairy cows offered a high forage TMR. However, the reduction in CH_4_ per unit of DMI relative to the control in the current study is lower than studies that offered 3-NOP in silage- and concentrate-based diets to lactating dairy cows (van Gastelen et al., 2022; −26 to −28%) and growing beef cattle (Kirwan et al., 2024; −27%). Discrepancies across studies may be partly due to differences in the chemical composition of the diets (Vyas et al., 2018; Kebreab et al., 2023). For instance, the diet offered to the animals in the current study was of a higher NDF content compared with van Gastelen et al. (2022) and Kirwan et al. (2024), which may have influenced the efficacy of 3-NOP. Vyas et al. (2018) postulated that NDF influences the rate at which CH_4_ is reduced by inhibitors such as 3-NOP, due to differences in the concentration of methyl-coenzyme M within the rumen. The dosage rate of 3-NOP is another factor reported to influence its efficacy (Kebreab et al., 2023). This is corroborated by Figure 2 in which variation in the measured dietary concentration of 3-NOP across the experimental weeks was moderately correlated with the efficacy of 3-NOP (R^2^ = 0.58; Figure 2). Variation in the measured dietary concentration of 3-NOP may be due to variation in mixing conditions within the mixing wagon or silage DM content over the experimental period (or both), which subsequently affected the efficacy of 3-NOP over time.

**Figure 2:**
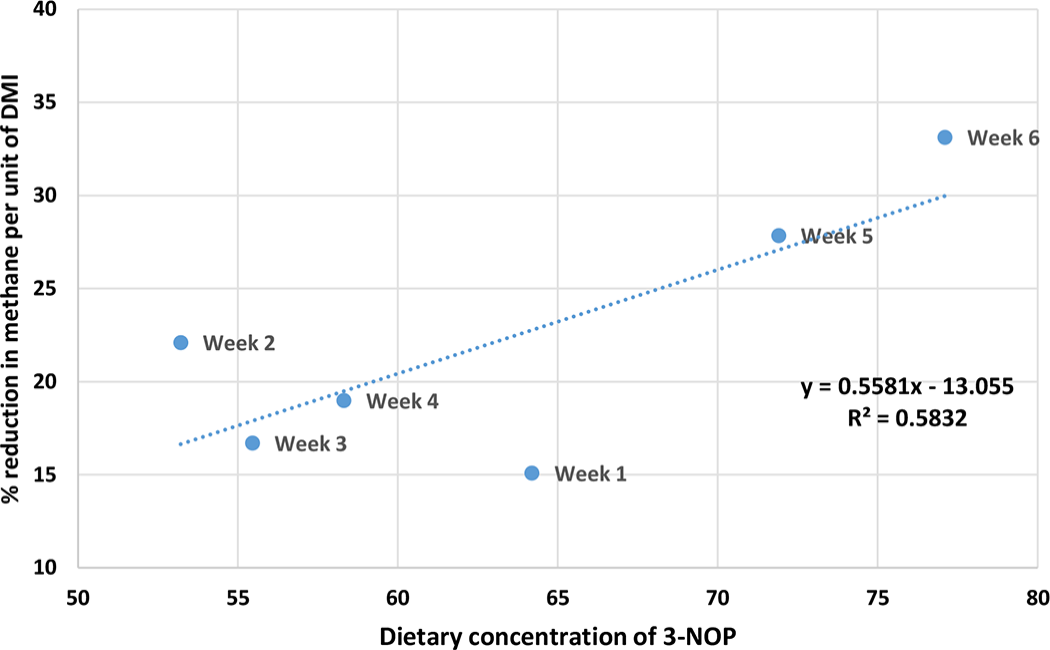
Graph showing the relationship between dietary 3-nitrooxypropanol (3-NOP) concentration and the percentage reduction in methane per unit of DMI of the treatment group relative to the control group across the experimental weeks.

The increased H_2_ output in cows supplemented with 3-NOP is in agreement with previous research (Hristov et al., 2015). The inhibition of enteric CH_4_ leads to an increase in H_2_ within the rumen fluid. However, only a proportion of this H_2_ is eructed as gaseous H_2_, with the majority redirected into alternative H_2_ sinks such as propionate (Hristov et al., 2015). Variations in H_2_ across the experimental period are unclear but may be due to variation in the partitioning of H_2_ to alternative sinks across the study period; however, this requires further research. Interestingly, these differences were unrelated to the CH_4_ abatement potential of 3-NOP across the study period. Previous studies have also noted that changes in H_2_ dynamics with 3-NOP supplementation over time are unrelated to reductions in CH_4_ output (Hristov et al., 2015; Melgar et al., 2020). Carbon dioxide levels were unaffected by 3-NOP supplementation, which is similar to the findings of Hristov et al. (2015) and suggests that there was no excessive enteric CO_2_ available after the inhibition of methanogenesis, which is potentially due to the CO_2_ being retained as absorbed energy.

In agreement with previous research (Hristov et al., 2015; van Gastelen et al., 2024), there was no impact of 3-NOP supplementation on DMI, BW, or BCS, while there was also no impact of 3-NOP supplementation on calf birth weight in the current study. This highlights the potential of 3-NOP to reduce methane output over the nonlactating period without implicating animal performance or calving characteristics. Nonetheless, the proportion of Irish dairy farmers in possession of mixer wagons capable of mixing additives throughout the forage may limit the ability of 3-NOP to deliver widespread reductions in enteric CH_4_ on pasture-based dairy farms during the winter housing period. Therefore, alternative methods of feeding the supplement, such as top dressing the material on grass silage, should also be explored.
